# Genome-wide identification of alcohol dehydrogenase (ADH) gene family under waterlogging stress in wheat (*Triticum aestivum*)

**DOI:** 10.7717/peerj.11861

**Published:** 2021-07-23

**Authors:** Changwei Shen, Jingping Yuan, Xingqi Ou, Xiujuan Ren, Xinhua Li

**Affiliations:** 1 School of Resources and Environmental Sciences, Henan Institute of Science and Technology, Xinxiang, Henan, China; 2 School of Horticulture and Landscape Architecture, Henan Institute of Science and Technology, Xinxiang, Henan, China; 3 School of Life Science and Technology, Henan Institute of Science and Technology, Xinxiang, Henan, China

**Keywords:** Alcohol dehydrogenase, *Triticum aestivum*, Expression patterns, Waterlogging stress

## Abstract

**Background:**

Alcohol dehydrogenase (*ADH*) plays an important role in plant survival under anaerobic conditions. Although some research about *ADH* in many plants have been carried out, the bioinformatics analysis of the *ADH* gene family from *Triticum aestivum* and their response to abiotic stress is unclear.

**Methods:**

A total of 22 *ADH* genes were identified from the wheat genome, and these genes could be divided into two subfamilies (subfamily I and subfamily II). All *TaADH* genes belonged to the Medium-chain ADH subfamily. Sequence alignment analysis showed that all TaADH proteins contained a conservative GroES-like domain and Zinc-binding domain. A total of 64 duplicated gene pairs were found, and the Ka/Ks value of these gene pairs was less than 1, which indicated that these genes were relatively conservative and did not change greatly in the process of duplication.

**Results:**

The organizational analysis showed that nine *TaADH* genes were highly expressed in all organs, and the rest of *TaADH* genes had tissue specificity. *Cis*-acting element analysis showed that almost all of the *TaADH* genes contained an anaerobic response element. The expression levels of *ADH* gene in waterlogging tolerant and waterlogging sensitive wheat seeds were analyzed by quantitative real-time PCR (qRT-PCR). This showed that some key *ADH* genes were significantly responsive to waterlogging stress at the seed germination stage, and the response of waterlogging tolerant and waterlogging sensitive wheat seeds to waterlogging stress was regulated by different *ADH* genes. The results may be helpful to further study the function of *TaADH* genes and to determine the candidate gene for wheat stress resistance breeding.

## Introduction

Alcohol dehydrogenase (ADH, EC1.1.1.1) is a zinc-binding enzyme that relies on NAD (P) cofactors to participate in the conversion between ethanol and acetaldehyde (Höög et al., 2003; Thompson et al., 2007). The *ADH* gene family is a large family, which is mainly divided into three subfamilies: short-chain dehydrogenase/reductase (SDR)-ADH (containing about 250 amino acid residues), medium-chain dehydrogenase/reductase (MDR)-ADH (containing about 350 amino acid residues) and long-chain dehydrogenase/reductase (LDR)-ADH (600~750 amino acid residues or about 385~900 amino acid residues) (Alka et al., 2013). (MDR)-ADH occupies a high proportion in the ADH gene family and usually contains zinc ligand in its activation site (Iaria et al., 2012; Min et al., 2012; Cheng et al., 2013). The (SDR)-ADH gene lacks zinc-liganding cysteine residues in their coenzyme binding regions (Kim et al., 2009; Strommer, 2011; Moummou et al., 2012) and the (LDR)-ADH gene has not been found in plants. ADH proteins usually contain the GroES-like domain (35~164 amino acids) and the zinc-binding domain (206~340 amino acids) (Murzin, 1996; Bhupesh & Mande, 1999).

ADH has been widely studied in human, animal, yeast and bacteria (Khan et al., 2010; Kumar et al., 2012; Çelik & Aktas, 2013; Jönvall et al., 2013; Plapp et al., 2013; Quaglia et al., 2013; Alka et al., 2013). Some reports have also been reported in plants (Jin et al., 2016; Zeng et al., 2020) and most plant *ADH* genes belong to (MDR)-ADH (Stephen & Klee, 2006). We found that *ADH* family genes from plants were usually involved in aroma synthesis during fruit development and were closely related to the formation of alcohols during fruit ripening (Van Der Straeten et al., 1991; Speirs et al., 1998; Echeverria et al., 2004; Moummou et al., 2012; Xu et al., 2014; Jin et al., 2016; Qin et al., 2017; Zeng et al., 2020). For instance, RNA-Seq and quantitative real-time PCR (qRT-PCR) analysis showed that the expression levels of 3 *ADH* genes (*Pbr013912.1*, *Pbr026289.1* and *Pbr01252.1*) in white pear were closely related to the content of aromatic compounds during fruit development (Zeng et al., 2020). *ADH* was also involved in the response to hormones. The *cis*-acting elements of the *CmADH* gene from *Pyrus bretschneideri* showed that *CmADH* genes were involved in roles of hormone regulation (Jin et al., 2016). The qRT-PCR analysis also showed that some *CmADH* genes had different response patterns to ABA, IAA and ethylene (Jin et al., 2016).

Since ADH activity is considered a necessary condition for plant survival under anaerobic conditions, the response of plant *ADH* gene to anaerobic stress has always been the research focus (Harberd, 1982; Liskens, 1966; Bailey-Serres & Voesenek, 2008). The transcriptional levels of *ADH1* and *ADH2* from *Zea mays* increased rapidly under hypoxia conditions at 6 h, but followed by a rapid decline at 12–18 h (Andrews, Cobb & Johnson, 1993). In *Arabidopsis thaliana*, the *AtADH* gene was continuously expressed in the root (including lateral roots) when the seedlings grew on the plate, but not in the shoot, which was similar to the expression pattern of *ADH1* in *Zea mays* (Zhang et al., 2010). Three *ADH* genes (*HvADH-1*, *HvADH-2*, and *HvADH-3*) in barley were significantly induced under anaerobic stress, and their activities were very high (Gonçalves et al., 2018). Studies on rice (Minhas, 1999), cucumber (Qi et al., 2012) and grape (Tesniere et al., 2006) also showed that plants could induce the expression of *ADHs* under hypoxia stress. Borrego-Benjumea et al. (2020) recently found the most significant changes in Gene Ontology (GO) terms, resulting from these differentially expressed genes (DEGs) observed under waterlogging stress in barley, were associated with the “hydrogen peroxide metabolic process”, “oxidation-reduction process” and “response to oxidative stress”.

Wheat is an important food crop widely planted globally, and its harvest area ranks first among the three major food crops (rice, wheat and maize). China is the world’s largest wheat producer and consumer; it is of great significance to China’s food security and increasing farmers’ income. According to the World Food and Agriculture Organization (FAO), about 10% of the world’s land area is affected by different degrees of waterlogging (Li et al., 2011). Wheat often encounters continuous rainy days during planting. Besides, uneven terrain or poor farmland drainage systems leads to stagnant water in the soil, which often leads to a lack of oxygen in seeds and roots, resulting in reduced wheat yield (Kırmızı & Bell, 2012). In the Mediterranean region, because about 40% of the annual rainfall occurs during the sowing period of winter wheat, the germination and growth stages of winter wheat are often affected by waterlogging (Bassu et al., 2009). Wheat-growing areas in south-central China also face the same problem (Arduini et al., 2016). It has been reported that *ADH* has high activity under anoxic conditions, and it mainly plays a role in converting acetaldehyde into alcohol in the last step of glycolysis or fermentation under anoxic conditions (Hageman & Flesher, 1960). At present, there are no systematic reports on the identification, structural characteristics, evolutionary relationship and response to waterlogging stress of *ADH* family genes in wheat. The release of the wheat genome (Appels et al., 2018) provides a basis for the identification and characteristic analysis of wheat *ADH* family genes. In this study, we identified 22 *ADH* genes from the wheat genome by bioinformatics methods. At the same time, we analyzed the distribution, physical and chemical characteristics, structural characteristics, gene duplication events and evolutionary relationship with other species of wheat *ADH* family genes. Additionally, we analyzed the tissue expression pattern of *TaADH* genes and their response to waterlogging stress. The above information will greatly promote our understanding of the functions of *TaADH* family genes.

## Materials & methods

### Identification of *ADH* gene family in wheat

The wheat genome data was downloaded from the wheat genome database (https://urgi.versailles.inra.fr/download/iwgsc/IWGSC_RefSeq_Assemblies/v1.0/) (Appels et al., 2018). We first used 26 melon ADH family protein sequences as search inquiry sequences and searched melon ADH homologous genes using the local BLAST program (amino acid identity > 70%, E value < 10^−10^). All melon ADH proteins were derived from the literature of Jin (Jin et al., 2016). Secondly, the ADH domain (PF00107.26, PF08240.12 and PF13602.6) was obtained from the Pfam database (http://pfam.xfam.org/) (Finn et al., 2010). We first used the HMMER3 software package to create the Hidden Markov Model file (Eddy, 2011), and then used HMMsearch with default parameters to search the wheat protein database. All the above candidate proteins were verified by Batch-CDD (https://www.ncbi.nlm.nih.gov/Structure/bwrpsb/bwrpsb.cgi) and SMART (http://smart.embl-heidelberg.de/) (Letunic, Doerks & Bork, 2012), and all proteins without GroES-like domain and zinc-binding domain were deleted.

The number of amino acids,protein molecular weight and isoelectric point of the candidate proteins were calculated by the ExPASy website (https://web.expasy.org/compute_pi/). Plant-mPLoc (http://www.csbio.sjtu.edu.cn/bioinf/plant-multi) (Fang et al., 2020) was used to predict the subcellular location of the *TaADH* genes.

### Evolutionary analysis of *TaADH* genes

To clarify the evolutionary relationship of ADH protein in wheat and the evolutionary relationship of ADH protein between wheat and several other species, all the ADH protein sequences were aligned by the ClustalW program and then used to constructed the phylogenetic tree by MEGA7.0 (Kumar, Stecher & Tamura, 2016). All the phylogenetic trees were constructed by the neighbour joining (NJ) method, 1000 replicates of bootstrap values, the pairwise deletion option. ADH protein sequences from *Arabidopsis thaliana*, *Cucumis melo*, *Cucumis sativus*, *Glycine max*, *Hordeum vulgare*, *Lycopersicon esculentum*, *Oryza sativa* and *Vitis vinifera* were downloaded from the NCBI (National Center for Biotechnology Information) database by using their gene IDs from the *Pyrus bretschneideri* reference (Jin et al., 2016).

### Analysis of structural characteristics of *TaADHs*

To clarify the structural characteristics of the *ADH* gene, the intron-exon distribution map of *TaADH* genes was generated by the Gene Structure Display Server 2.0 (GSDS2.0, http://gsds.gao-lab.org/) (Hu et al., 2015). The generation of the intron-exon distribution map depends on the cDNA sequence and the corresponding genomic DNA sequence of the wheat ADH gene.

To clarify the domain of wheat ADH protein, we used DNAMAN software to carry out the multiple sequence alignment of all TaADH protein sequences. Besides, we used Multiple EM for Motif Elicitation (MEME, http://meme-suite.org/tools/meme) (Bailey et al., 2006) to analyze the motif of TaADH proteins. The program was set according to the following parameters: the optimal width of motif is 650 amino acid residues, and the maximum number of motifs is 15. Finally, the motifs map of TaADH protein were presented by TBtools (https://github.com/CJ-Chen/TBtools) (Chen et al., 2020).

### Distribution of *TaADHs* on chromosomes and gene duplication events

To determine the position of *TaADHs* on the chromosome, the starting position of *TaADHs* was extracted from the Chinese Spring wheat genome (https://urgi.versailles.inra.fr/download/iwgsc/IWGSC_RefSeq_Assemblies/v1.0/) and finally presented by TBtools (Chen et al., 2020).

To identify the *TaADH* gene duplication events, the open reading frame of all *TaADHs* were BLAST compared with each other by the local BLASTN program (Identity > 80%, e-value < 1e^−10^). Gene alignment coverage was then acquired using the previously calculated method: gene alignment coverage = (alignment length-mismatch length)/the length of larger genes (Wang et al., 2010). When the gene alignment coverage was more than 0.75, they were considered to be a duplicated gene (Wang et al., 2010). Besides, in the 100 kb region, two duplicated genes separated by other genes were considered as tandem duplicated genes. When the distance between two duplicated genes was more than 100 kb or the duplicated genes were distributed on different chromosomes, they were named as fragment duplicated genes. Non-synonymous substitution rate (Ka), Synonymous substitution rate (Ks) and Ka/Ks were calculated by DnaSP software (http://www.ub.edu/dnasp/) (Rozas et al., 2003). The formula: T = Ks/2λ × 10^−6^ Mya was used to calculate divergence time (T), λ = 6.5 × 10^−9^ represented the rate of divergence of synonymous substitutions per site per year, the unit of evolution time was millions of years (Mya) (Emanuelsson et al., 2000).

### Analysis of tissue expression pattern of *TaADHs*

To analyze the expression pattern of TaADH genes in root, stem, leaf, spike and grain, we downloaded the RNA-seq reads data from Chinese Spring wheat through expVIP (an expression visualization and integration platform) platform (http://www.wheat-expression.com/). Raw data was deposited as DRP000768 and SRP028357 (https://urgi.versailles.inra.fr/files/RNASeqWheat/) on NCBI and were derived from the Chinese Spring (123 non-stressed samples, 15 tissues) (Borrill, Ramirezgonzalez & Uauy, 2016). TPM (transcripts per kilobase of exon model per million mapped reads) values were used as expression units on expVIP software. After obtaining the average TPM expression level of all TaADH genes, the heatmap was drawn with TBtools software (https://github.com/CJ-Chen/TBtools) (Chen et al., 2020).

### *Cis*-acting element analysis of *TaADH* genes

To analyze the *cis*-acting element of *TaADH* genes, the promoter sequences (2,000 bp before the start codon) of all *TaADH* genes were extracted from the Chinese Spring wheat database. The *cis*-acting elements of these genes were predicted by online tool PLANTCARE (https://bioinformatics.psb.ugent.be/webtools/plantcare/html/) (Lescot et al., 2002) and visualized by TBtools (https://github.com/CJ-Chen/TBtools) (Chen et al., 2020).

The functional enrichment analysis was performed using g:Profiler (version e102_eg49_p15_7a9b4d6) (https://biit.cs.ut.ee/gprofiler/gost) with g:SCS multiple testing correction method applying significance threshold of 0.05 (Raudvere et al., 2019). The 22 TaADH gene IDs were uploaded into the program, and ‘*Triticum aestivum*’ was chosen as the reference organism to analyze molecular function, cellular components, and biological processes.

### Expression analysis of *TaADHs* under waterlogging stress

To study the waterlogging tolerance of *TaADHs*, ‘Zhoumai 22’ (ZM22) and ‘Bainong 607’ (BN607) were used as materials in this study. The materials were cultivated and provided by professor Ou’s team of the School of Life Science and Technology of Henan Institute of Science and Technology.

Experimental group: 50 seeds of each variety were submerged in the 14-cm-diameter glass Petri dish and filled with 200 mL of sterilized deionized water (pH 6.8; electrical conductivity 1.5 μS cm^−1^), and enveloped in aluminium foil to minimize gas exchange at 20 °C in the dark for 3 days. The seeds were germinated for 24 and 72 h (cultured in a Petri dish containing a layer of filter paper and 10 mL of aseptic deionized water) after waterlogging treatment. Control group: the seeds of each variety germinated normally without waterlogging treatment. All the Petri dishes containing seeds were placed in a growth chamber with 25 °C, 75% relative humidity, 16 h light/8 h dark cycle. The experimental group and the control group were repeated five times. The characteristics of ZM22 and BN607 were identified by phenotypic analysis and bud length measurement between the experimental group and the control group.

To determine the response of *TaADH* genes to waterlogging, we used the qRT-PCR to determine the expression level of *ADHs*. The seeds were collected at 24 and 72 h after waterlogging stress (the buds and roots of the seeds were removed), and the RNA was extracted by RNAsimple Total RNAKit (Tiangen, Beijing, China). To avoid the contamination of genomic DNA, RNase-free DNase I (Takara, Tokyo, Japan) was used to remove DNA from the total RNA. The first-strand cDNAs were synthesized using the PrimeScript RT Reagent Kit with gDNA Eraser (Takara, Dalian, China) according to the manufacturer’s protocol. The qRT-PCR assays were performed with the Primer Script RT Reagent Kit (Takara, Dalian, China) and 18S (*AJ272181.1*) was used as a reference gene. The quantitative primers of all *TaADH* genes were designed by Primer 6.0 software, and BLAST searched against the wheat database to determine the specificity of primers (Table S1). The PCR conditions were as follows: 95 °C for 10 s and 40 cycles of 95 °C for 5 s and 60 °C for 30 s. The qRT-PCR was performed using an ABI Step One Plus. All the experiments were performed with three biological replicates. The relative expression was calculated using the 2^−ΔΔCt^ method (Livak & Schmittgen, 2001).

## Results

### Genome-wide identification and physicochemical characteristics of *ADH* genes in *Triticum aestivum*

A total of 22 *ADH* genes were identified in the wheat genome based on the BLAST program. According to the location distribution of these genes on chromosomes (from Ta1A, Ta1B, Ta1D to Ta7A, Ta7B, Ta7D, from top to bottom), they were named *TaADH1*~*TaADH22* (Table 1, Table S2). The length of predicted coding sequences of 22 *TaADH* genes ranged from 1,044 to 1,244 bp, the number of corresponding amino acids ranged from 347 to 415 aa. The theoretical isoelectric point (*PI*) ranged from 5.68 to 8.2, and the molecular weight ranged from 34.4 to 44.2 kDa. Through the subcellular localization prediction of *TaADH* genes, it was found that they were all localized to the cytoplasm.

**Table 1:** Properties and locations of the predicted TaADH proteins in *T. aestivum*.

Gene ID	Gene name	Ta_Chr^a^	Start^b^	End^c^	CDs length (bp)	Number of amino acid	Isoelectric point (*pI*)^d^	Molecular weight (Mw) KDa^e^	Subcellular localization^f^
TraesCS1A02G370100.1	*TaADH1*	1A	547391832	547395833	1143	380	6.08	41.6	Cytoplasm.
TraesCS1A02G370200.1	*TaADH2*	1A	547410788	547417410	1137	378	5.87	40.8	Cytoplasm.
TraesCS1B02G389200.1	*TaADH3*	1B	622706402	622708971	1469	379	6.28	41.1	Cytoplasm.
TraesCS1D02G376300.1	*TaADH4*	1D	452625185	452627671	1484	379	6.03	41.1	Cytoplasm.
TraesCS4A02G202100.2	*TaADH5*	4A	491715851	491719316	1140	379	6.15	41.0	Cytoplasm.
TraesCS4A02G202200.1	*TaADH6*	4A	491914927	491917719	1430	379	5.81	40.9	Cytoplasm.
TraesCS4A02G202300.1	*TaADH7*	4A	492029965	492032871	1718	379	5.97	41.0	Cytoplasm.
TraesCS4B02G106300.1	*TaADH8*	4B	115556136	115560148	1962	379	6.03	41.0	Cytoplasm.
TraesCS4B02G106400.1	*TaADH9*	4B	115845355	115848000	1348	376	5.91	40.5	Cytoplasm.
TraesCS4B02G106500.1	*TaADH10*	4B	115879177	115881956	1611	379	5.9	34.4	Cytoplasm.
TraesCS4D02G103000.1	*TaADH11*	4D	81918232	81921969	1839	379	6.15	41.0	Cytoplasm.
TraesCS4D02G103100.1	*TaADH12*	4D	81971499	81974375	1467	379	5.92	40.9	Cytoplasm.
TraesCS4D02G103300.1	*TaADH13*	4D	81984987	81987448	1044	347	6.56	37.6	Cytoplasm.
TraesCS5A02G193900.1	*TaADH14*	5A	397249660	397251898	1359	365	5.68	39.7	Cytoplasm.
TraesCS5B02G189200.1	*TaADH15*	5B	341062699	341068539	1140	379	5.83	40.9	Cytoplasm.
TraesCS5D02G196300.2	*TaADH16*	5D	299832208	299835185	1751	379	5.68	41.0	Cytoplasm.
TraesCS6A02G386600.1	*TaADH17*	6A	603279456	603282956	1367	381	6.55	40.6	Cytoplasm.
TraesCS6B02G425700.1	*TaADH18*	6B	694401891	694405637	1529	381	6.37	40.7	Cytoplasm.
TraesCS6D02G371200.1	*TaADH19*	6D	456554723	456558968	1629	381	6.37	40.7	Cytoplasm.
TraesCS7A02G322200.1	*TaADH20*	7A	466247186	466249779	1248	415	8.2	44.0	Cytoplasm.
TraesCS7B02G223100.1	*TaADH21*	7B	419035483	419038096	1248	415	8.18	44.2	Cytoplasm.
TraesCS7D02G319100.1	*TaADH22*	7D	407849007	407852341	2011	415	8.2	44.1	Cytoplasm.

**Notes:**

a Ta_Chr,The chromosome name.

b Start, Predicted starting position of mRNA.

c End, Predicted termination position of mRNA.

d *pI*, Theoretical Isoelectric point.

e MW, Molecular weight (Mw) predicted by ExPASy (http://web.expasy.org/tools/).

f Subcellular location of the TaADH proteins was predicted by Plant-mPLoc (http://www.csbio.sjtu.edu.cn/bioinf/plant-multi).

### Classification and conserved domain analysis of 22 TaADHs

**Figure 1:**
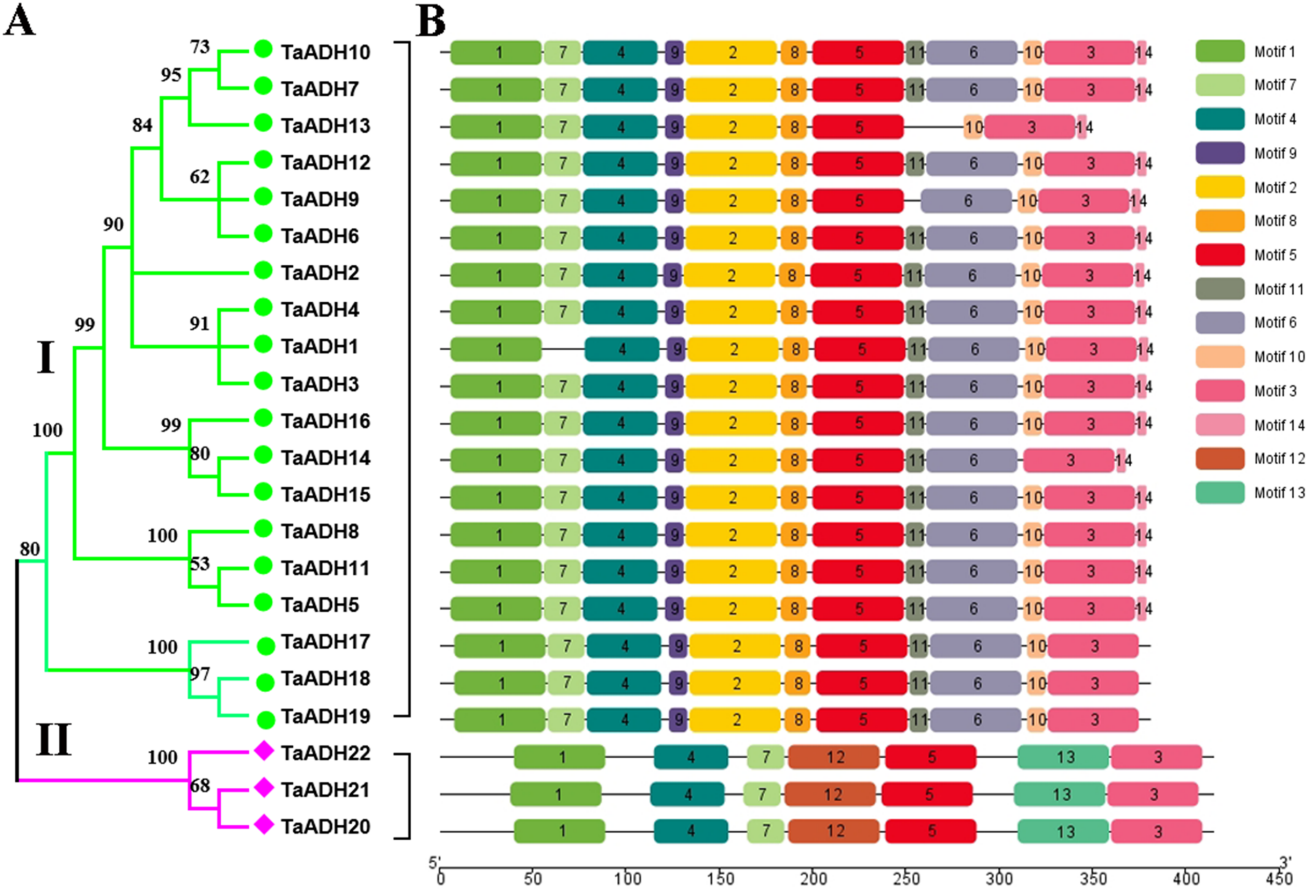
Phylogenetic relationship and motif analysis of ADH proteins in wheat. (A) An unroot phylogenetic tree of 22 TaADH proteins. (B) The motif composition of TaADH proteins. There were a total of 14 motifs, each of which was represented by a specific number.

To analyze the evolutionary relationship of 22 TaADH proteins, the phylogenetic tree was constructed by MEGA7.0. According to the amino acid sequence identity, 22 TaADH proteins were divided into two subfamilies (subfamily I, subfamily II) (Fig. 1A). Subfamily I contained the largest number of (10) TaADH proteins, and the remaining three TaADH proteins (TaADH20~22) belonged to subfamily II.

To analyze the conserved motif of TaADH protein, we used MEME online software to analyze the 22 TaADH protein sequences. A total of 14 motifs were detected in the wheat ADH family (Fig. 1B, Fig. S1). Motif 1, 3, 4 and 5 existed in all TaADH proteins, indicating that these TaADH proteins were highly conserved (Fig. 1B). Motif 2, 8, 9 and 11 existed only in subfamily I, while motif 12, 13 only existed in subfamily II (Fig. 1B), indicating structural differences and specificity among different subfamilies. Motif 7 was only missed in TaADH1, motif 11 was only missed in TaADH13, TaADH9 and subfamily II, and motif 10 was only missed in TaADH14 and subfamily II.

Comparing the sequences of 22 TaADH proteins, all of these protein sequences contained conserved GroES-like domain and Zinc-binding domain (Fig. S2). GroES-like domain contained 35~164 amino acids, and the Zinc-binding domain contained 206~340 amino acids, which correspond to the structural characteristics of the *ADH* gene family.

### Exon-intron analysis of 22 *TaADHs*

**Figure 2:**
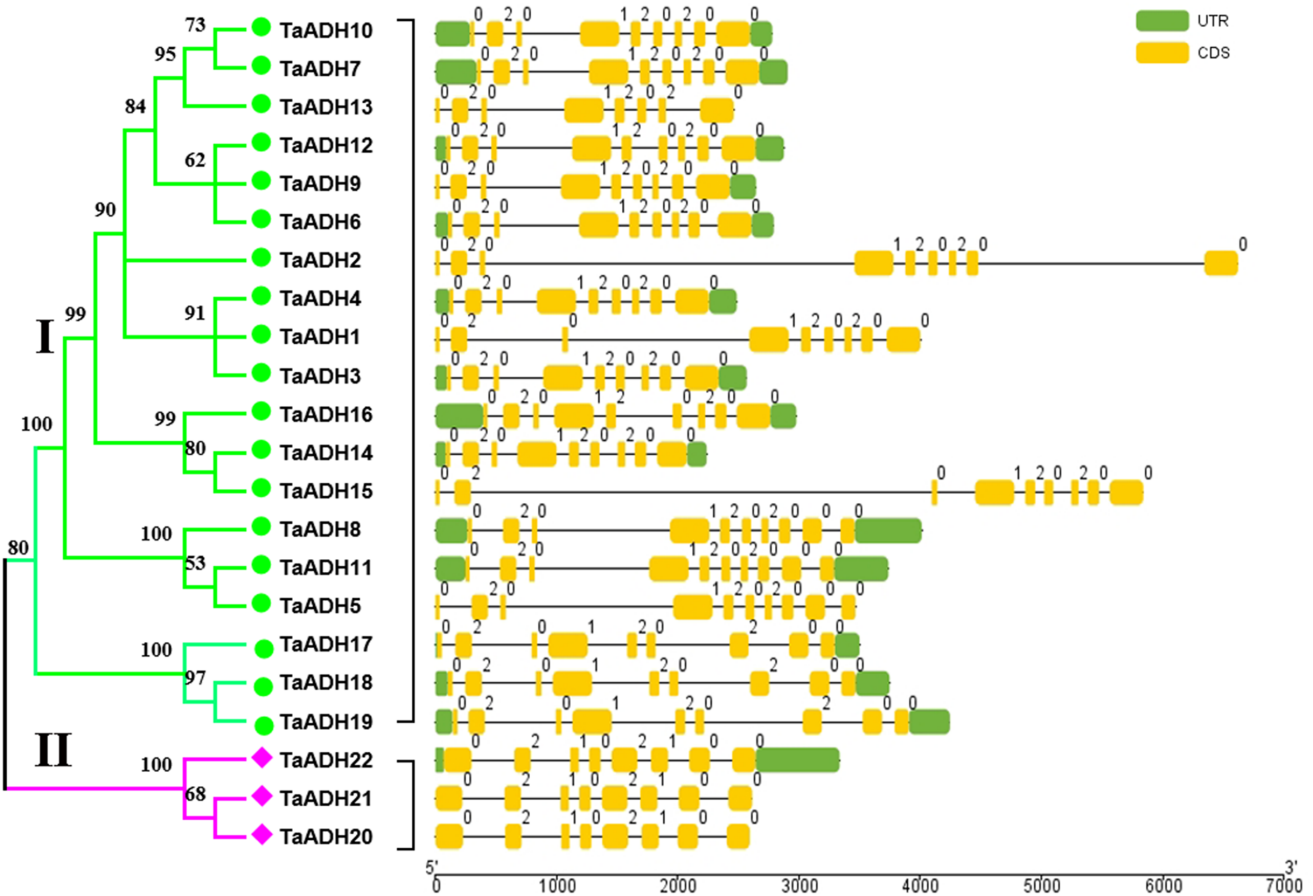
Intron-exon structure analysis of the *ADH* gene in wheat. The exons, introns and untranslated regions (UTRs) were indicated by yellow boxes, black lines and green boxes, respectively. The size of exons and introns can be estimated from the bottom scale.

Through the analysis of intron-exon structure, we found that 22 *TaADH* genes contained 8~10 exons (Fig. 2). In subfamily I, three genes (*TaADH5*, *TaADH8* and *TaADH11*) contained six exons, two genes (*TaADH13* and *TaADH16*) contained eight exons, and the remaining *TaADH* genes contained nine exons (Fig. 2). In subfamily II, all *TaADH* genes contained eight exons (Fig. 2). As a whole, we found that the same branch contained similar structural patterns.

### Chromosomal distribution and gene duplication of 22 *TaADHs*

**Figure 3:**
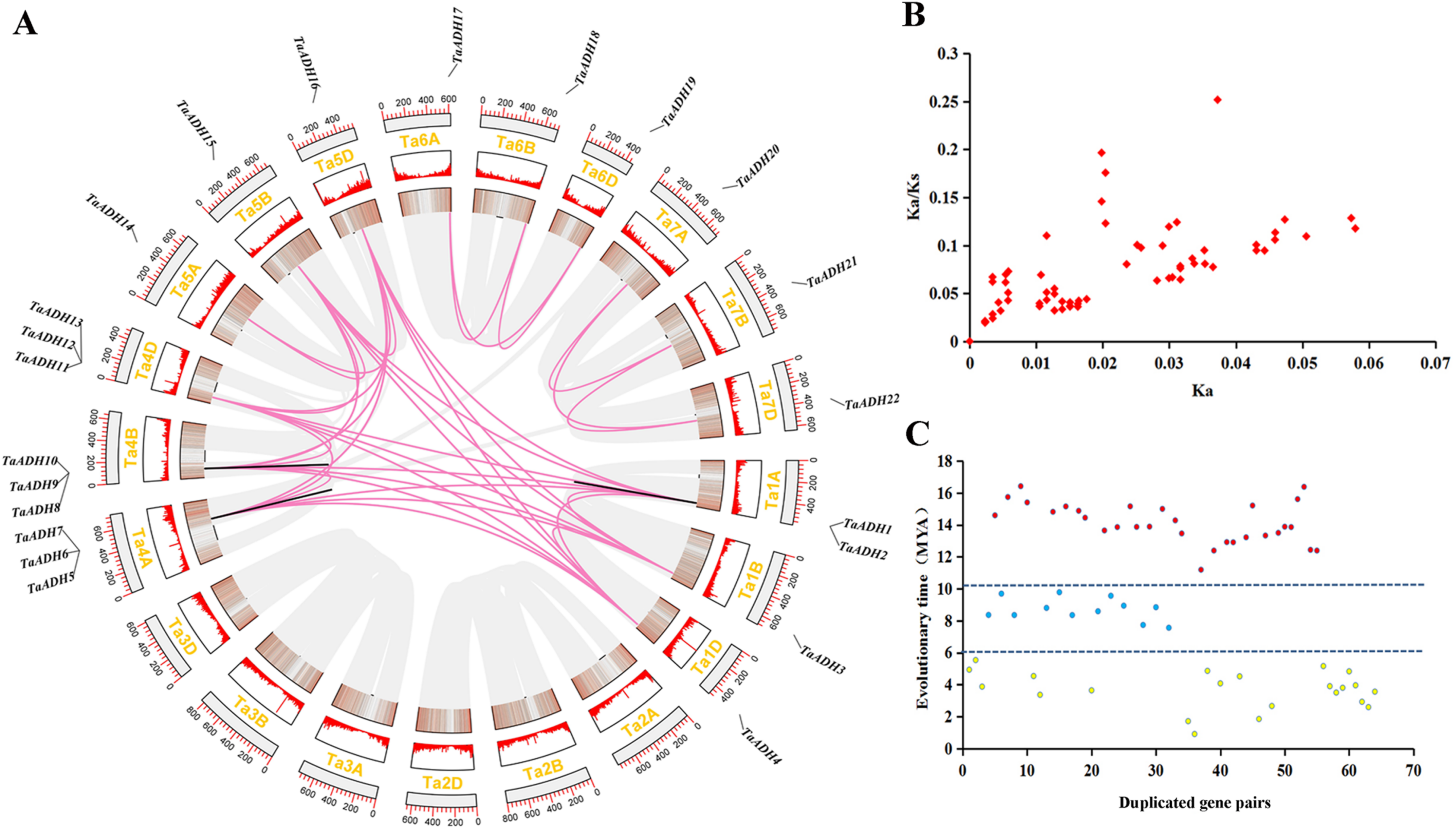
Location distribution of *TaADH* genes on chromosomes and analysis of duplicated genes. (A) Distribution of 22 *TaADH* genes on chromosomes and duplicated gene pairs in wheat. Red lines represented of duplicated genes pairs on different chromosomes, while black lines represented duplicated genes pairs on the same chromosome. (B) The Ka/Ks of 64 duplicated genes pairs. (C) The evolutionary time of 64 duplicated genes pairs. Ka, the non-synonymous substitution rate; Ks, synonymous substitution rate; Mya, million years ago.

According to the initial position of 22 *TaADHs* on the chromosome, they were visualized by TBtools. Twenty-two TaADHs were unevenly distributed on 15 of 21 chromosomes (they do not exist on chromosomes Ta2A-D and Ta3A-D) (Fig. 3A). There were three genes on chromosomes Ta4A-D, respectively; two genes on chromosome Ta1A; and one gene on the other chromosomes, respectively (Fig. 3).

By analyzing the duplication events of 22 *TaADHs*, we found 64 pairs of duplicated genes (Fig. 3, Table S3). Most of these duplicated genes were on different chromosomes, *TaADH1*-*TaADH2*, *TaADH1*-*TaADH3*, *TaADH6*-*TaADH7* and *TaADH9*-*TaADH10* were on the same chromosome, respectively. However, we found that only the distances of *TaADH1*-*TaADH2* and *TaADH9*-*TaADH10* were less than 100 kb, indicating that the two duplicated gene pairs had tandem duplication events, and the remaining duplicated gene pairs had undergone fragment duplication events (Table 1, Table S3).

To reveal the selection pressure of *TaADH* family genes in the process of evolution, the non-synonymous substitution rate (Ka), synonymous substitution rate (Ks) and Ka/Ks for 64 duplicated pairs were calculated (Fig. 3B, Table S3). All Ka/Ks of these duplicated pairs were less than 1, which tended to a pure selection, indicating that the sequence similarity of *TaADH* genes was very high and relatively conservative in the process of evolution. The evolution time of the duplicated events of *TaADH* genes can be divided into three evolution periods (Fig. 3C, Table S3). The first period was 11.19~16.42 million years ago (Mya), with 30 duplicated gene pairs. Twelve TaADH duplicated gene pairs occurred at 7.56~9.56 Mya. The remaining 22 TaADH duplicated gene pairs occurred at 0.9~5.53 Mya. Although these gene sequences were conserved, they were different in evolutionary time.

### Phylogenetic relationship of *ADHs* in *Triticum aestivum* and *A. thaliana*

**Figure 4:**
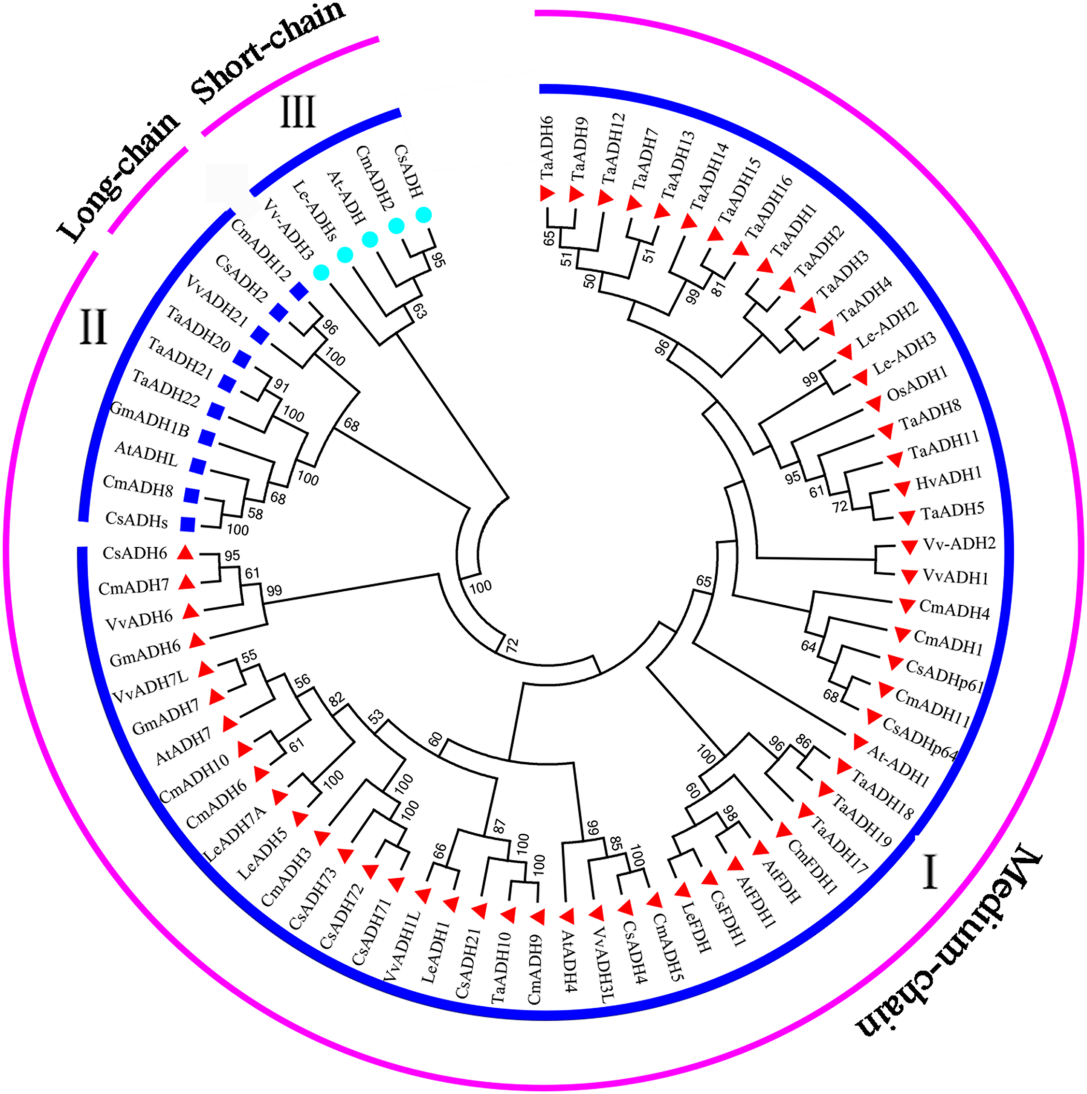
The evolutionary relationship between TaADHs and other nine species. Eighty percent cut-off value was used for the condensed tree. At, *Arabidopsis thaliana*; Cm, *Cucumis melo*; Cs, *Cucumis sativus*; Hv, *Hordeum vulgare*; Os, *Oryza sativa*; Gm, *Glycine max*; Le, *Lycopersicon esculentum*; Ta, *Triticum aestivum*; Vv, *Vitis vinifera*.

To study the evolutionary relationship between *Triticum aestivum* (22) *Arabidopsis thaliana* (7), *Cucumis melo* (13), *Cucumis sativus* (12), *Glycine max* (3), *Hordeum vulgare* (1), *Lycopersicon esculentum* (7), *Oryza sativa* (1) and *Vitis vinifera* (8), a phylogenetic tree was constructed. According to the amino acid sequence identity, all ADH proteins were divided into three subfamilies (subfamily I, II, III) (Fig. 4), which was consistent with the classification of TaADH in Fig. 1A. TaADHs existed only in subfamily I and subfamily II. Subfamily I contained the most (59) ADH proteins, subfamily III contained the least (5) ADH proteins, and the remaining proteins (10) belonged to subfamily II. Besides, according to the number of amino acid residues, all ADH proteins in subfamily I belonged to medium-chain ADH proteins, 3 ADH proteins from subfamily II belonged to long-chain-ADH, the remaining proteins from subfamily II belonged to medium-chain ADH proteins (Fig. 4). All TaADHs belonged to medium-chain ADH protein (Fig. 4).

The same subfamily usually contained ADH proteins of several species. For instance, subfamily II contained ADH proteins from *Arabidopsis thaliana*, *Cucumis melo*, *Cucumis sativus*, *Glycine max*, *Triticum aestivum* and *Vitis vinifera*, which indicated that these species came from the same ancestor a long time ago.

### Tissue expression patterns of *TaADH* genes

**Figure 5:**
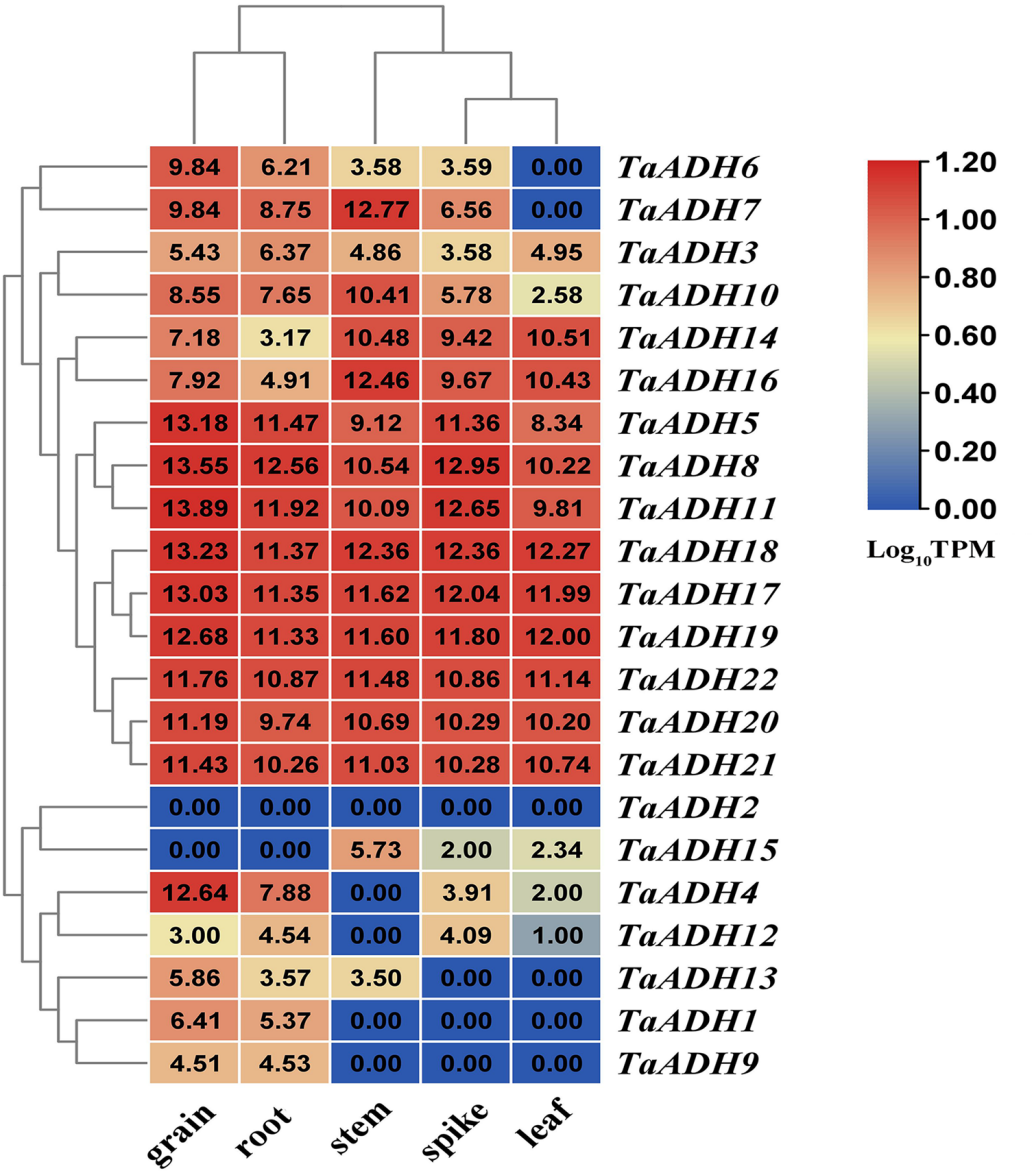
Distribution and statistical analysis of *cis*-acting elements in *TaADHs* promoter. The number of squares represented the predicted number of *cis*-acting elements. The colour scale on the right also represented the number of *cis*-acting elements. The darker the red shading was, the higher the number.

According to the transcriptome data of different wheat tissues, we analyzed the tissue expression pattern of 22 *TaADH* genes in roots, leaf, stem, spike and grain (Fig. 5). Nine genes (*TaADH8*, *TaADH11*, *TaADH18*, *TaADH17*, *TaADH19*, *TaADH5*, *TaADH22*, *TaADH20* and *TaADH21*) were highly expressed in all tissues. Six genes (*TaADH12*, *TaADH13*, *TaADH1*, *TaADH9*, *TaADH2* and *TaADH15*) were not expressed or low expressed in all tissues; the remaining genes were only highly expressed in specific tissues, such as *TaADH4* and *TaADH6* were highly expressed in grain, but not or low expressed in other tissues.

For the duplicated genes, we found that they had the same tissue expression patterns, such as *TaADH8*_*TaADH11*, *TaADH17*_*TaADH18*, *TaADH20*_*TaADH21*, and so on. There were also differences in the expression patterns of some other duplicated genes (Fig. 5). For example, in the duplicated gene pair *TaADH9*_*TaADH16*, *TaADH9* was only expressed in grain and root. In contrast, *TaADH16* was expressed in all tissues, and the expression profiles in grain and root were lower than that in other tissues (Fig. 5), so it was speculated that duplicated genes had been diversified in the process of evolution.

### The *cis*-regulatory element analysis and GO annotation of *TaADH* genes

**Figure 6:**
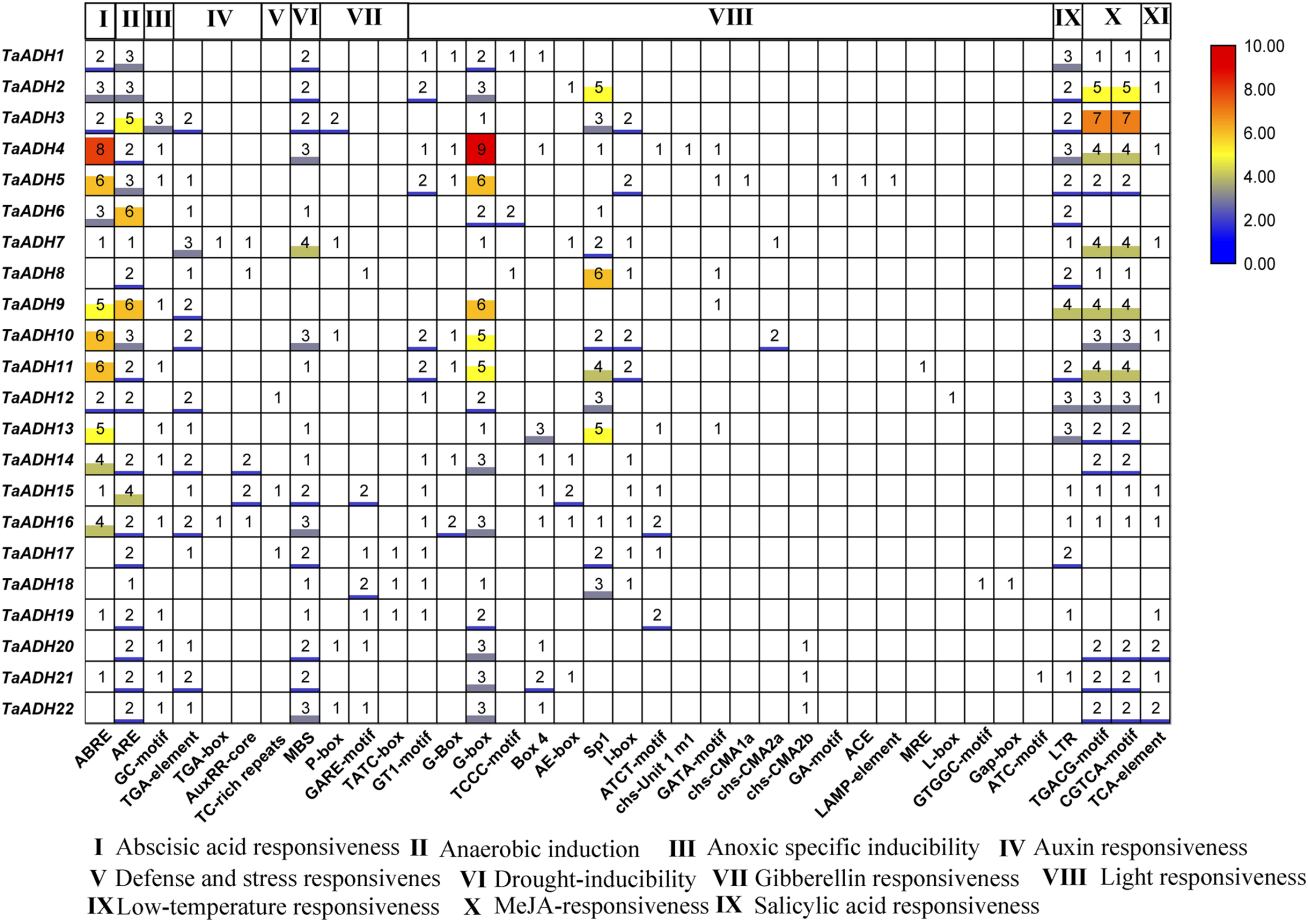
The expression of *TaADH* genes in various tissues. A heatmap was constructed based on the TPM data (http://www.wheat-expression.com/) using TBtools software. The number on the heat map represented the TPM value, and the colour scale represented the range of Log_10_TPM. In the colour scale, dark red shading indicates higher values, and dark blue shading indicates lower values. The lower the value, the bluer.

To analyze the potential function of 22 TaADH genes, we analyzed the cis-acting elements of *TaADH* gene promoters. Through the analysis of 2,000 bp before the start codon, we found that a total of 563 cis-acting elements, which responded to 11 stresses (biotic and abiotic), including hormone response, anaerobic response, defence, and stress response, drought induction, light response, low-temperature response, etc. (Fig. 6, Table S4). Seventeen genes (77%) respond to ABA, of which the *TaADH4* gene contained eight abscisic acid responsiveness elements (ABREs), which suggested that the gene may play a key role in the abscisic acid response. The TGACG-motif and CGTCA-motif were methyl jasmonate response elements (Chen & Qiu, 2020). *TaADH3* each contained seven TGACG-motifs and seven CGTCA-motifs, so it was speculated that this gene played a key role in methyl jasmonate response. Anaerobic response element (ARE) contained a conserved AAACCA sequence, mainly involved in anaerobic induction. In this study, we found that except for *TaADH13* did not contain ARE, all other *TaADH* genes contained ARE elements. For example, *TaADH6* and *TaADH9* contained six AREs, respectively, which suggested that *TaADH* family genes may play key roles under anaerobic stress.

By analyzing the functional characteristics of *TaADH* genes, namely ‘molecular function’, ‘biological process’, and ‘cellular components’, it can help us understanding the function of proteins at the molecular level. For the category of molecular function (Fig. S3, Table S5), in addition to *TaADH1* (*TraesCS1A02G370100.1*), other 21 *TaADH* genes were involved 15 GO categories, and they were all associated with “zinc ion binding” (GO:0008270), “oxidoreductase activity” (GO:0016491), “metal ion binding” (GO:0046872), “cation binding” (GO:0043169), “ion binding” (GO:0043167) and “catalytic activity” (GO:0003824). *TaADH3* (*TraesCS1B02G389200.1*) ~ *TaADH8* (*TraesCS4B02G106300.1*) and *TaADH11* (*TraesCS4D02G103000.1*) were involved in “alcohol dehydrogenase activity” (GO:0004022) and “oxidoreductase activity” (GO:0016491). From the perspective of ‘biological process’ categories, all *TaADHs* participated in 30 GO categories, and they were associated with “oxidation-reduction process (GO:0008152)” and “metabolic process (GO:0055114)”. *TaADH4* (*TraesCS1D02G376300.1*) ~ *TaADH8* and *TaADH11* genes participated in 24 GO categories. Moreover, *TaADH17* (*TraesCS6A02G386600.1*) ~ *TaADH19* (*TraesCS6D02G371200.1*) were involved with “ethanol metabolic process (GO:0006067)”, “ethanol oxidation (GO:0006069)”, “primary alcohol metabolic process (GO:0034308)” and “alcohol metabolic process (GO:0006066)”. The ‘cell components’ annotation predicted the *TaADH4* ~ *TaADH8* and *TaADH11* existed in cytosol (GO:0005829).

### Phenotypic analysis of ZM22 and BN607 under waterlogging stress

**Figure 7:**
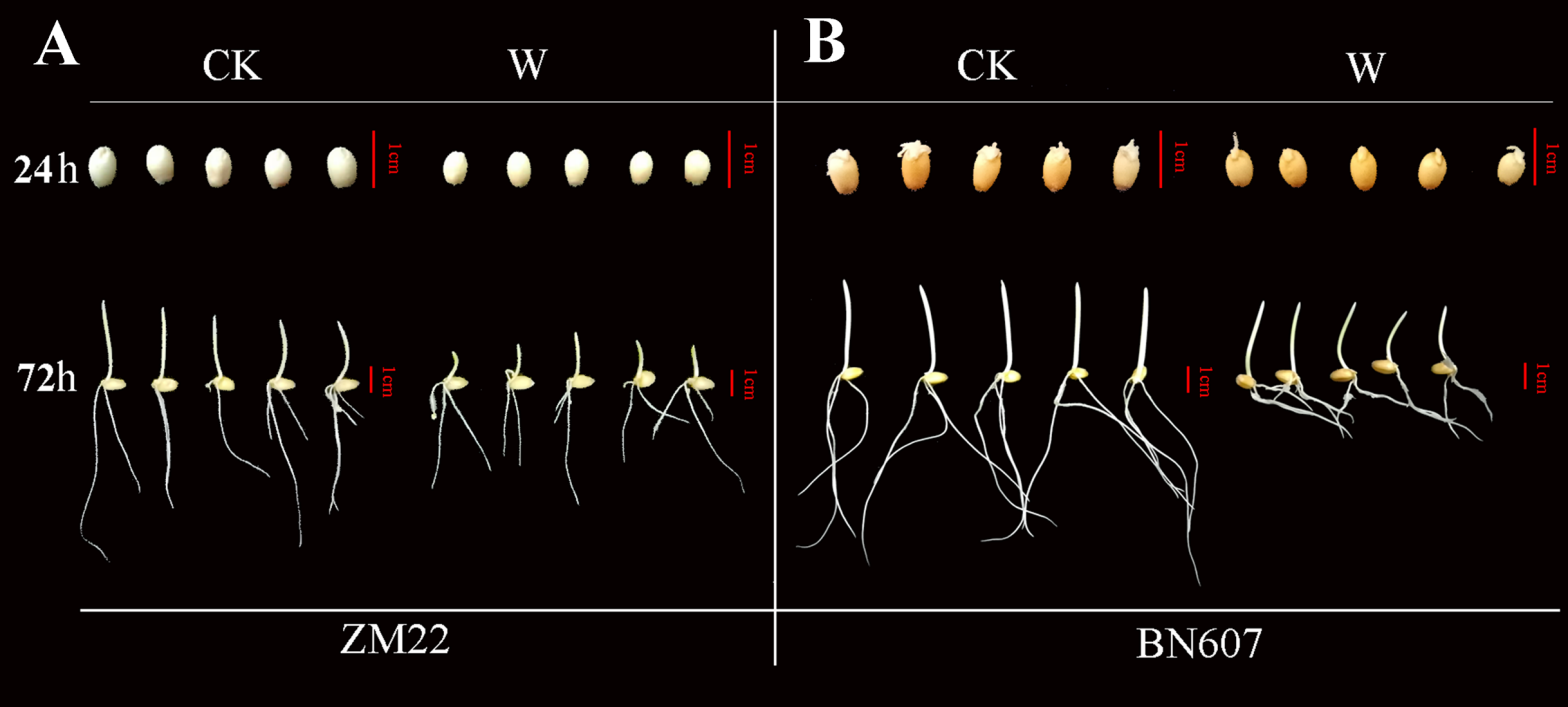
Phenotypic analysis of two kinds of wheat seeds germinated for 24 h and 72 h after waterlogging. The phenotype of ZM22 was on the left (A), and the phenotype of BN607 was on the right (B). CK, without waterlogging treatment; W, waterlogging treatment. The scale was 1 cm.

*ADH* gene plays a key role in plants under anaerobic conditions (Bailey-Serres & Voesenek, 2008). To study the expression of the *ADH* gene (Fig. 7, Table S6) in wheat seeds at 24 and 72 h after waterlogging for 3 days, we selected the seeds of two wheat varieties (Zhoumai 22 (ZM22) and Bainong 2008 (BN607)) for analysis.

At 24 h after the waterlogging treatment, the bud length of ZM22 and BN607 had no significant difference with the control seeds (no waterlogging treatment). However, all the seeds of ZM22 and BN607 germinated at 72 h after the waterlogging treatment. The bud length of ZM22 was significantly lower than that of the control seeds, while the bud length of BN607 was not significantly different from that of the control seeds (Fig. 7, Table S6), which further indicated that BN607 was more tolerant to waterlogged stress than ZM22.

### Expression analysis of *TaADH* genes under waterlogging treatment

**Figure 8:**
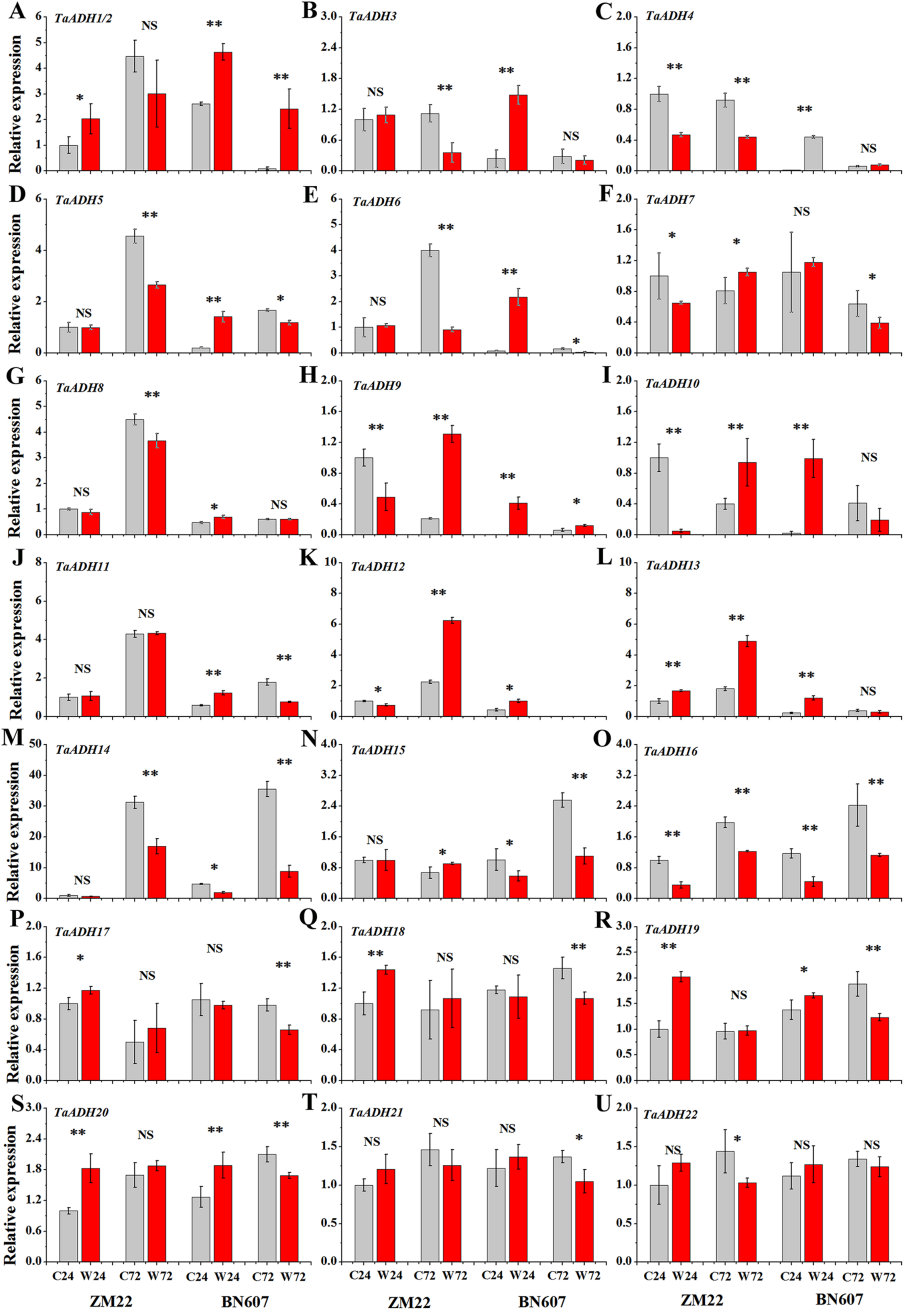
The relative expression profile of *TaADH* genes (A-U) in seeds of two wheat varieties under waterlogging stress. ZM22: Zhoumai 22, BN607: Bainong 607; C24: The seeds were directly germinated for 24 h without waterlogging treatment; W24: After being waterlogged, the seeds germinated for 24 h; C72: The seeds were directly germinated for 72 h without waterlogging treatment; W72: After being waterlogged, the seeds germinated for 72 h; each value represents the average of three biological repetitions. The error bars represented the SDs. An asterisk (*) indicates significance at *p* < 0.05, two asterisks (**) indicates significance at *p* < 0.01, NS indicates no significance.

The expression profiles of 22 *TaADH* genes in ZM22 and BN607 seeds at 24 and 72 h after waterlogging and control treatment were analyzed (Fig. 8, Table S6). The results showed that in ZM22, the expression of *TaADH1*/*2*, *TaADH13*, *TaADH17*, *TaADH18*, *TaADH19* and *TaADH20* was significantly up-regulated at 24 h of germination. However, among these genes, only the *TaADH13* gene was significantly induced at 72 h after waterlogging. There was no significant difference in the relative expression level of *TaADH1*/*2*, *TaADH17*, *TaADH18*, *TaADH19* and *TaADH20* between the waterlogging and control treatment at 72 h after waterlogging.

In BN607, the expression of *TaADH1*/*2*, *TaADH3*-*6*, *TaADH8*-*13*, *TaADH19* and *TaADH20* was significantly up-regulated at 24 h after waterlogging. Besides, *TaADH1*/*2*, *TaADH3* and *TaADH9* genes were also significantly induced at 72 h after waterlogging. Moreover, the relative expression levels of *TaADH5*, *TaADH6*, *TaADH14* and *TaADH16* were significantly lower than that of the control at 72 h after waterlogging (Fig. 8, Table S6). Based on the above analysis, we found that the response of waterlogging-sensitive ZM22 and waterlogging-tolerant BN607 to waterlogging stress at different times mainly depended on the time-specific expression of some key *ADH* genes in wheat.

## Discussion

In recent years, with global warming, extreme weather occurs more frequently, in which flood disaster is one of the abiotic stresses faced by plants, hypoxia will first occur in the flooded environment. Under waterlogging stress, plants often undergo lactic acid fermentation, resulting in cell acidification. To avoid cytoplasmic acidosis, ethanol production is necessary for plants to survive under anaerobic conditions (Roberts et al., 1984). Ethanol dehydrogenase plays a key role in converting ethanol and acetaldehyde (Sasnauskas et al., 1992; Gutheil, Holmquist & Vallee, 1992; Hjelmqvist et al., 1995). With the development of sequencing technology, the genomes of many species can be analyzed, which promotes the identification of the plant *ADH* gene family at the genome-wide level. *ADH* family genes from tomato (Moummou et al., 2012), rice (Kitaoka et al., 2016), barley (Kasbauer et al., 2018), *Cucumis melon* L. (Jin et al., 2016) and *Pyrus bretschneideri* (Qin et al., 2017) have been detected. Considering the disclosure of the wheat genome, it is possible to systematically identify the *TaADH* family members.

In this study, 22 *ADH* genes were identified in the wheat genome. *Arabidopsis thaliana ADH1* and *ADH3* contained 6 and 4 introns, respectively, while *ADH* in Chinese cabbage contained five introns (Strommer, 2011). However, there were 7~9 introns in *TaADH* genes, which was consistent with the number of introns in *ADH* genes from barley (Strommer, 2011). For example, *ADH2*/*3* in barley and *TaADH* both contained eight introns.

Plant *ADH* family were usually divided into short-chain ADH and medium-chain ADH. There was no report on long-chain ADH in plants (Kitaoka et al., 2016). One short-chain alcohol dehydrogenase/reductase SDRs (*OsMAS/SDR110C-MS1*) was found in rice (Thompson et al., 2007; Borras et al., 2014). Twenty-two *TaADHs* identified in this study belonged to medium-chain-ADH. These genes have highly conserved functional domain (GroES-like domain and zinc-binding domain) (Fig. S2), which was similar to the structure of *PbrADHs* in *Pyrus bretschneideri* (Qin et al., 2017), *VvADH2* in *Vitis vinifera* L. (Tesnière & Verriès, 2000), *CmADH1* in *Cucumis melon* L. (Manríquez et al., 2006) and *AtADH1* in *Arabidopsis thaliana* (Cheng et al., 2013).

Gene duplication is an important evolutionary process of gene family expansion, and gene duplication provides an opportunity for functional differentiation. Functional differences caused by gene duplication are considered to be important factors in species formation and environmental adaptability (Salse et al., 2008; Arsovski et al., 2015; Das et al., 2016; Laha et al., 2020). Therefore, the analysis of duplicated genes can help us to better understand the evolution of genes and species. In this study, we found that there were 64 duplicated gene pairs in the TaADHs family, all of which belong to (MDR)-ADH. Their Ka/ Ks values were all less than 1 (Fig. 3B), which means that all duplicated gene pairs were purified.

The *Ks* value was used to estimate the evolution time when duplication events occurred. The results showed that the duplicated events of *TaADH* family genes occurred between 0.90~16.42 Mya. The evolution time of 30 duplicated gene pairs was 11.19~16.42 Mya, and the evolution time of 12 duplicated gene pairs was 7.56~9.56 Mya, which was earlier than the first time of wheat genomic duplication (Ling et al., 2013).

Gene promoters are important factors in regulating gene expression patterns. They regulate gene expression at the transcriptional and post-transcriptional levels (Higo et al., 1998). *Cis*-acting elements in a specific promoter region participated in tissue-specific expression patterns under various environmental conditions. There was a positive correlation between the number of *cis*-acting elements and the degree of stimuli (Higo et al., 1998). In this study, some *cis*-acting elements of stress response, such as ABRE, ARE, MBS, LTR, TGACG-motif and CGTCA-motif, appeared in the promoter region of *TaADH* genes. They are involved in abscisic acid response, hypoxia induction, drought induction, low temperature response and methyl jasmonate response. 22 *TaADH* genes had at least one *cis*-acting element related to stress response, indicating its potential function in response to abiotic stress. We found that 21 *TaADH* genes (except *TaADH13*) were induced by waterlogging stress, and the promoters of all genes contained a *cis*-acting element for anaerobic induction (ARE). An anaerobic response complex (ARC) was found in maize, which consists of 5′ -GC (G/C) CC-3′ ( GC ) and 5′ -GGTTT-3′ (GT) components. It was an essential element for activation of the *ADH1* promoter in maize and Arabidopsis under anaerobic induction (Olive et al., 1990; Dolferus et al., 1994). It was found in rice that under waterlogging stress, the *Sub1a* gene of the ERF family in rice leaves could delay leaf senescence by regulating hormones, and the decrease in ABA content was due to the decrease in the expression of ABA biosynthesis-related genes (Garg et al., 2013; Saha et al., 2021 ). We also found that the promoter regions of 17 *TaADH* genes contained ABRE elements, indicating that plants would also participate in the adaptive response to environmental stress through a series of hormones (e. g., ethylene and ABA) under waterlogging stress.

There are several scenarios of flooding that provoke oxygen deficiency: waterlogging, which leads to local root hypoxia or anoxia (develops due to the activity of soil bacteria, plant root systems, and other soil biota), as well as complete flooding of the plant (submergence), which is often the cause of total anoxia (Gupta, Zabalza & Van Dongen, 2009). Under waterlogging or submergence, plants are exposed to a reduction in oxygen (O_2_) supply because of the slow diffusion rate of O_2_ in water and its limited solubility. Under anaerobic conditions, glycolysis, as well as alcoholic and lactic acid fermentation, are stimulated (Shikov, Chirkova & Yemelyanov, 2020). *ADH* has a good protective effect on hypoxia stress, seed development and aerobic metabolism of pollen after flooding (Bailey-Serres & Voesenek, 2008; Macnicol & Jacobsen, 2001). *ADH* activity may play an important role in the process of seed germination under hypoxia stress (Strommer, 2011). There are many kinds of *ADH* isozyme genes in seeds. Through tracking the activity of *ADH* isozyme during seed development, we found that *ADH* isozyme genes have activity at different times. Three *ADH* genes (*HvADH1*, *HvADH2* and *HvADH3*) were found in barley. The activity of *HvADH1* could be detected during aerobic growth. Hypoxia can induce the expression of *HvADH1* and *HvADH2*, and the expression level of *HvADH-3* was significantly lower than that of *HvADH1* and *HvADH2* under hypoxia (Kasbauer et al., 2018). Pistelli et al. (2012) found that the expression patterns of *ADH* gene in different between the roots of plum rootstock S. 4 with waterlogging tolerance and the roots of wild type (WT) under waterlogging. Under hypoxia, the transcription level of *ADH3* in WT roots did not change, while the transcription level of *ADH1* in WT roots was significantly higher than that under oxygen supply. The expression levels of the two genes in the root of plum rootstock S.4 under hypoxia treatment were significantly lower than that of the control treatment. Interestingly, this study also found that the expression patterns of *TaADH7* and *TaAHD11* genes in ZM22 and BN607 varieties at 72 h after waterlogging treatment were consistent with the expression patterns of *ADH1* and *ADH3* genes in rootstock WT and S.4 clones of plum rootstock. There were some differences in response patterns of *ADH* gene expression among different plants under short-term and long-term waterlogging stress. Transcriptional expression of *OsADH1* gene in 5-day-old rice seedlings reached the highest level at 24 h under short-term 36 h waterlogging stress (Manangkil, Rafael & Nakamura, 2019). The *GmAdh2* gene that responded to flooding was isolated from soybean cultivar Enrei. The expression of *GmAdh2* was significantly increased 6 h after flooding and decreased 24 h after floodwater drainage (Komatsu et al., 2011). When *Taxodium hybrid* ‘Zhongshanshan 406’ of two-year-old was completely submerged in water, the expression levels of *ThADH1* and *ThADH4* in the roots were increased and the elevated expression pattern were continued until 50th day, and their expression levels in the roots reached 63 and 23 times of those on 0 day, respectively (Xuan et al., 2021). In this study, under short-term waterlogging stress, the *TaADH* genes in two varieties with different waterlogging tolerance had different expressions patterns at different times. The functions of these genes need to be further verified by experiments.

The functional annotation enrichment showed that most *TaADHs* were enriched in the oxidoreductase activity and oxidation-reduction process (Fig. S3). The function of these genes was closely related to the differential expression of *TaADHs* in two different waterlogging-tolerant varieties under waterlogging stress. We also found that *TaADH3*, *TaADH5*, *TaADH8*, *TaADH10-11*, *TaADH14* and *TaADH16-22* were expressed in all parts of wheat, which suggested that these genes may be constantly expressed. In rice seedlings, *ADH1* and *ALDH2a* genes were significantly induced under flooding stress, but decreased rapidly after flooding, indicating that the high expression of *AHD1* and *ALDH2a* may be one reason why rice is more tolerant to flooding than other plants (Manangkil, Rafael & Nakamura, 2019). When wheat seeds were exposed to an anaerobic environment (waterlogging treatment), the expression of *TaADHs* varied with the exposure time. No matter which cultivar studied, Bainong 607 or Zhoumai 22, the expression levels of *TaADH1*/*2*, *TaADH13*, *TaADH19* and *TaADH20* genes were significantly higher than those of the control 24 h after waterlogging treatment (Fig. 8). Tissue expression analysis showed that the relative expression of these genes was relatively high in grains, especially *TaADH1* and *TaADH13*, which suggested that these genes may play a key role in grains under anaerobic stress. At 72 h after waterlogging treatment, the relative expressions of *TaADH1*/*2*, *TaADH3* and *TaADH9* genes in BN607 were significantly higher than in control. However, only the *TaADH13* gene was highly expressed in Zhoumai 22 seeds 72 h after waterlogging treatment (Fig. 8), so it was speculated that *TaADH1*/*2*, *TaADH3* and *TaADH9* play an important role in waterlogging stress and are an important basis for screening waterlogging tolerant wheat varieties.

## Conclusions

A total of 22 *TaADH* genes were identified in the wheat genome. These genes were distributed on 15 chromosomes. All of the TaADH protein sequences contained the GroES-like domain and Zinc-binding domain. Through phylogenetic tree analysis with other species, it was found that 22 *TaADH* genes in wheat belonged to medium-chain ADH type and were grouped into two subfamilies. There were 64 duplicated gene pairs, and they experienced purification selection. *TaADH8*, *TaADH11*, *TaADH18*, *TaADH17*, *TaADH19*, *TaADH5*, *TaADH22*, *TaADH20* and *TaADH21* were highly expressed in all tissues, and the remaining *TaADH* genes had tissue-specific. *Cis*-acting elements analysis showed that 22 *TaADH* genes were responsive to 11 kinds of abiotic stress, 21 of which responded to anaerobic stress. By comparing the expression profiles of waterlogging-tolerant wheat Bainong 607 and waterlogging-intolerant wheat Zhoumai 22 at the germination stage after waterlogging treatment, some key candidate genes of wheat waterlogging-tolerant were identified. These results will provide valuable information regarding further functional elucidation of *TaADH* genes in wheat.

## Supplemental Information

10.7717/peerj.11861/supp-1 Supplemental Information 1 List of primer sequences used for qRT-PCR analysis.

10.7717/peerj.11861/supp-2 Supplemental Information 2 The coding sequence and protein sequence for 22 TaADHs.

10.7717/peerj.11861/supp-3 Supplemental Information 3 Ka/Ks calculation and estimated divergence time for the *TaADH* duplicated gene pairs.

10.7717/peerj.11861/supp-4 Supplemental Information 4 Detailed information about *cis* -acting elements of *TaADH* gene promoter.

10.7717/peerj.11861/supp-5 Supplemental Information 5 List of Gene Ontology (GO) in *TaADH* genes.

10.7717/peerj.11861/supp-6 Supplemental Information 6 The relative expression level of *TaADH* genes under waterlogging stress and the determination of bud length of two kinds of wheat seeds after 72 h of germination.

10.7717/peerj.11861/supp-7 Supplemental Information 7 The logos of 15 motifs in 22 TaADH proteins.

10.7717/peerj.11861/supp-8 Supplemental Information 8 The amino acid sequences alignment of TaADH proteins. Sequence alignment was performed using the DNAMAN5.0 program. The GroES-like domain and zinc-binding domain were labelled with green and black, respectively.

10.7717/peerj.11861/supp-9 Supplemental Information 9 Gene Ontology (GO) analysis in *TaADH* genes. The GO enrichment analyses were performed by g:Profiler (version e102_eg49_p15_7a9b4d6) (https://biit.cs.ut.ee/gprofiler/gost) with g:SCS multiple testing correction method applying significance threshold of 0.05. The adjusted *p*-values of the enrichment significant were transformed by −log10.

## Additional Information and Declarations

### Data Availability

The following information was supplied regarding data availability: Raw data are available in the Supplemental Files.
