# Supplementary figures and images for: Genome-wide identification of alcohol dehydrogenase (ADH) gene family under waterlogging stress in wheat (Triticum aestivum)
Source: PeerJ. 2021 Jul 23;9:e11861. doi: 10.7717/peerj.11861 (PMC8312495; doi:10.7717/peerj.11861)

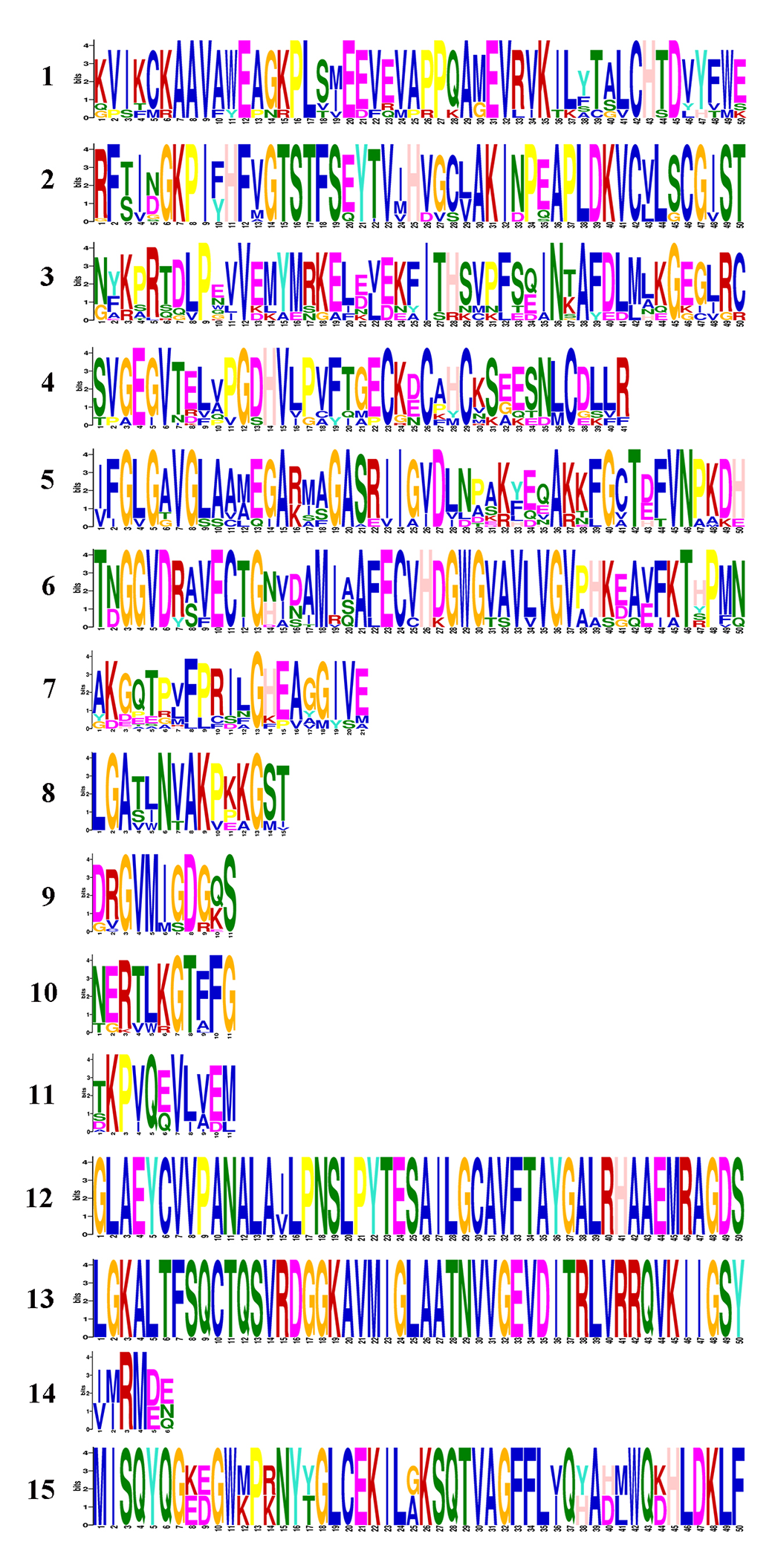

Supplement: Supplemental Information 7 [file peerj-09-11861-s007.png]

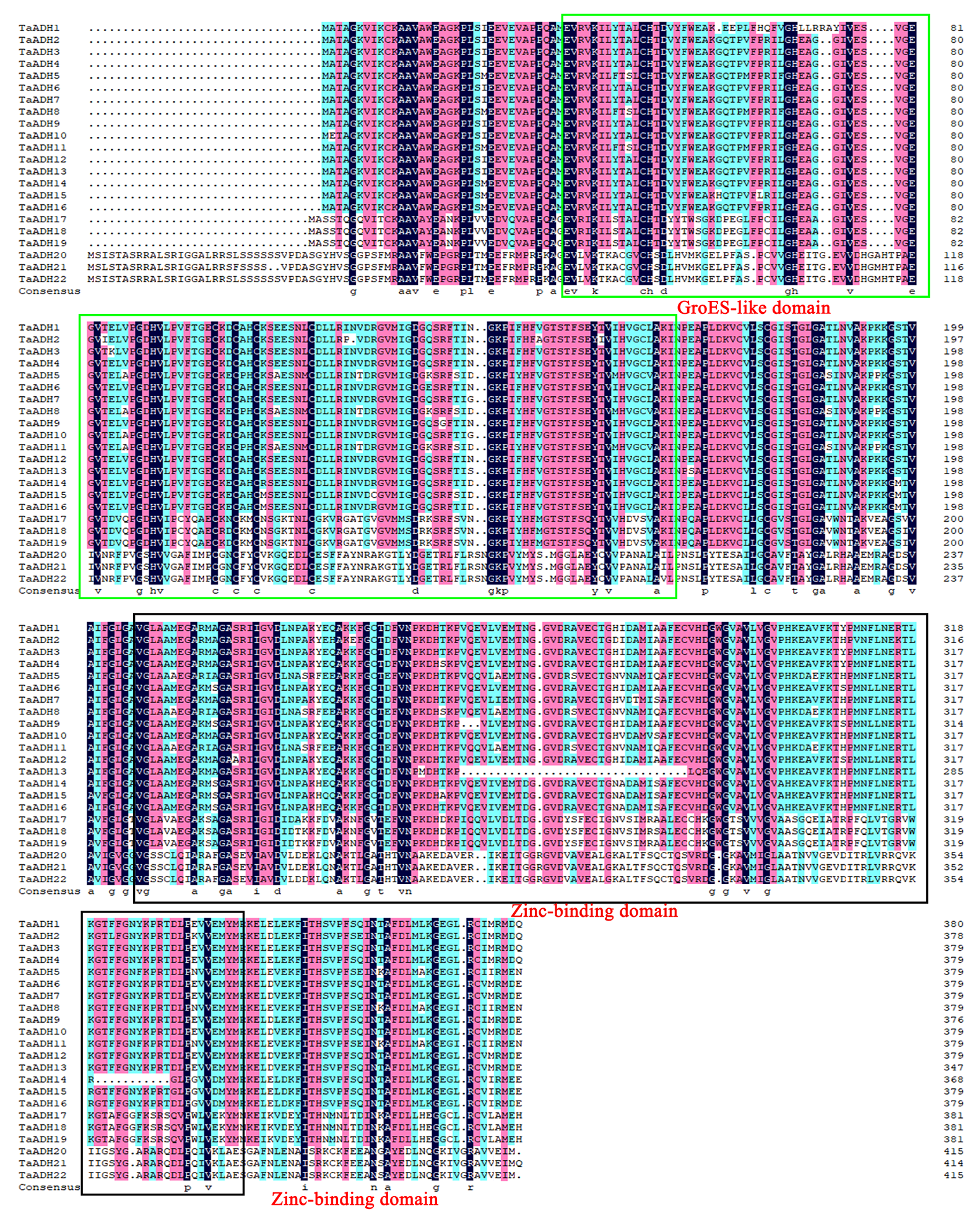

Supplement: Supplemental Information 8 — The GroES-like domain and zinc-binding domain were labelled with green and black, respectively. [file peerj-09-11861-s008.png]

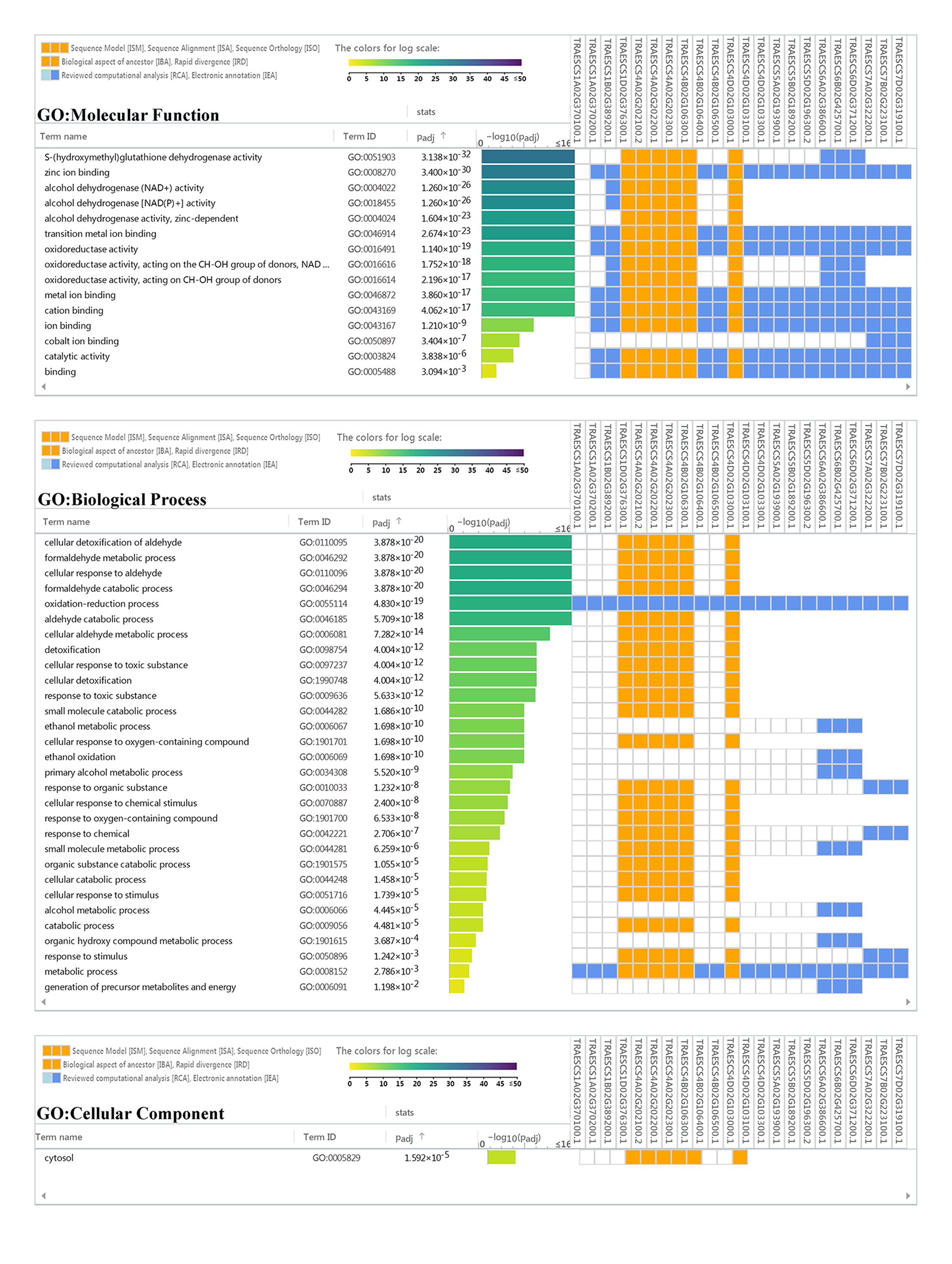

Supplement: Supplemental Information 9 — The GO enrichment analyses were performed by g:Profiler (version e102_eg49_p15_7a9b4d6) (https://biit.cs.ut.ee/gprofiler/gost) with g:SCS multiple testing correction method applying significance threshold of 0.05. The adjusted p-values of the enrichment significant were transformed by −log10. [file peerj-09-11861-s009.png]
